# Simultaneous identification of robust synergistic subnetwork markers for effective cancer prognosis

**DOI:** 10.1186/s13637-014-0019-9

**Published:** 2014-11-06

**Authors:** Navadon Khunlertgit, Byung-Jun Yoon

**Affiliations:** 1 Department of Electrical and Computer Engineering, Texas A&M University, College Station, 77843-3128 TX USA; 2 College of Science, Engineering, and Technology, Hamad Bin Khalifa University (HBKU), Doha, P.O. Box 5825 Qatar

**Keywords:** Cancer classification, Subnetwork marker identification, Protein-protein interaction network, Message-passing algorithm

## Abstract

**Background:**

Accurate prediction of cancer prognosis based on gene expression data is generally difficult, and identifying robust prognostic markers for cancer remains a challenging problem. Recent studies have shown that modular markers, such as pathway markers and subnetwork markers, can provide better snapshots of the underlying biological mechanisms by incorporating additional biological information, thereby leading to more accurate cancer classification.

**Results:**

In this paper, we propose a novel method for simultaneously identifying robust synergistic subnetwork markers that can accurately predict cancer prognosis. The proposed method utilizes an efficient message-passing algorithm called affinity propagation, based on which we identify groups – or subnetworks – of discriminative and synergistic genes, whose protein products are closely located in the protein-protein interaction (PPI) network. Unlike other existing subnetwork marker identification methods, our proposed method can simultaneously identify multiple nonoverlapping subnetwork markers that can synergistically predict cancer prognosis.

**Conclusions:**

Evaluation results based on multiple breast cancer datasets demonstrate that the proposed message-passing approach can identify robust subnetwork markers in the human PPI network, which have higher discriminative power and better reproducibility compared to those identified by previous methods. The identified subnetwork makers can lead to better cancer classifiers with improved overall performance and consistency across independent cancer datasets.

**Electronic supplementary material:**

The online version of this article (doi:10.1186/s13637-014-0019-9) contains supplementary material, which is available to authorized users.

## 1 Introduction

Identifying disease-related biological markers is an important problem in translational genomics, and there have been significant research efforts to find robust markers for disease diagnosis and prognosis from gene expression data obtained from microarrays or next-generation sequencing (NGS). However, the small sample size and the high dimensionality of the typical genomic data makes the prediction of such biomarkers very challenging. A large number of approaches have been proposed so far to deal with these issues, where it has been recently shown that the concept of ‘modular markers’ have potentials for detecting better disease markers that are more robust and reproducible across independent datasets. In the past, it has been a common practice to look for the so-called ‘key genes’ that show significant differential expression under different conditions or between distinct phenotypes to discover gene markers that may be used for discriminating between different classes of biological/clinical samples. Unlike these traditional gene markers, where each gene is viewed as a potential biomarker, a *modular marker* consists of multiple genes that belong to the same functional module and show coordinated behaviors to fulfill a common biological function. The utilization of modular markers allows us to interpret and analyze the gene expression data in a more system-oriented way, which may facilitate the prediction of system-level properties based on the markers.

Examples of such modular markers include the pathway markers [1]-[5] and the subnetwork markers [6],[7]. A pathway marker consists of multiple genes that belong to the same functional pathway. In order to use a pathway marker in a classification task, we first need to infer the activity level of the pathway based on the expression levels of its member genes, after which the inferred pathway activity can be used as a feature in a classifier. So far, several different methods have been proposed for pathway activity inference [1]-[5], and it has been shown that pathway markers tend to be more effective and robust compared to traditional gene markers. Unfortunately, the usefulness of pathway markers is practically limited by our incomplete pathway knowledge. In fact, currently known pathways cover only a relatively small number of genes; hence, the reliance on pathway markers may result in excluding crucial genes that may play important roles in determining the phenotypes of interest.

The concept of subnetwork markers has been originally proposed to address the weakness of pathway markers [6],[8]. The main idea is to overlay the protein-protein interaction (PPI) network with the gene expression data to identify potential ‘subnetwork markers,’ which consist of discriminative genes whose protein products interact with each other, hence, connected in the PPI network. Conceptually, we can find such subnetwork markers by identifying subnetwork regions that undergo significant differential expression across different phenotypes, and the detected subnetwork markers may potentially correspond to functional modules – such as signaling pathways or protein complexes – in the underlying biological network. PPI networks provide a much better gene coverage compared to the set of currently known pathways; hence, this network-based approach can essentially overcome the major shortcoming of the pathway-based approach.

Until now, several different strategies have been proposed for identifying subnetwork markers. For example, Chuang et al. [6] proposed an efficient algorithm for finding subnetwork markers, where they first identify highly discriminative seed genes and then greedily grow the subnetworks around the seed genes to maximize the mutual information between the average *z*-score of the member genes and the class label. More recently, Su et al. [7] proposed a different strategy, where differentially expressed linear paths are found by dynamic programming and overlapping paths are combined to obtain discriminative subnetwork markers. Both studies [6],[7] have shown that subnetwork markers can lead to more accurate and robust classifiers, compared to pathway markers.

In this paper, we propose a novel method for identifying effective subnetwork markers for predicting cancer prognosis. The proposed method is based on an efficient message-passing algorithm, called affinity propagation, which can be used to efficiently identify clusters of discriminative and synergistic genes whose protein products are either connected or closely located in the PPI network. Unlike previous subnetwork marker identification methods, the proposed method can simultaneously predict multiple subnetwork markers, which are mutually exclusive and have the potential to accurately predict cancer prognosis in a synergistic manner. Based on several independent breast cancer datasets, we demonstrate that the proposed method can identify better prognostic markers that have improved reproducibility and higher discriminative power compared to the markers identified by previous methods.

## 2 Materials and methods

### 2.1 Datasets

We obtained four independent breast cancer microarray gene expression datasets from previous studies, which we refer to as the USA dataset (GEO:GSE2034) [9], Netherlands dataset (NKI-295) [10], Belgium dataset (GEO:GSE7390) [11], and Sweden dataset (GEO:GSE1456) [12], respectively. The USA, Belgium, Sweden datasets were profiled on the Affymetrix U133a platform and downloaded from the Gene Expression Omnibus (GEO) website [13]. The Netherlands dataset was profiled on a custom Agilent microarray platform, and it was downloaded from the Stanford website [14]. The USA dataset contains the gene expression profiles of 286 breast cancer patients, the Netherlands dataset contains the profiles of 295 patients, the Belgium dataset contains the profiles of 198 patients, and the Sweden dataset contains the profiles obtained from 159 patients. In this study, gene expression profiles of the patients for whom metastasis had been detected within 5 years of surgery were labeled as ‘metastatic’, while the remaining profiles were labeled as ‘non-metastatic’. The USA, Netherlands, Belgium, and Sweden datasets respectively contain 106, 78, 35, and 35 metastatic profiles. The human protein-protein interaction network used in this paper was obtained from a previous study on subnetwork marker identification by Chuang et al. [6], which consists of 11,203 proteins and 57,235 interactions. We overlaid the gene expression data in the four breast cancer datasets with this PPI network, by mapping each gene to the corresponding protein in the network. After removing the proteins that do not have corresponding genes in all four datasets, we obtained an induced network with 26,150 interactions among 4,936 proteins.

### 2.2 The affinity propagation algorithm: a brief overview

In order to identify discriminative subnetwork markers, we apply *affinity propagation*[15], an efficient clustering algorithm based on a message-passing approach. In affinity propagation, real-valued messages are iteratively exchanged between data points until a good set of exemplars (i.e., representative data points) are identified. The data points are clustered around the exemplars that best represent them, which gives rise to clusters that consist of similar data points. During the message-passing process, two different types of messages are exchanged between data points: *responsibility* and *availability*. The responsibility *r*(*i*,*k*) measures the suitability of the data point *k* to be an exemplar of the data point *i*, considering other potential exemplars. The availability *a*(*i*,*k*) measures the appropriateness of choosing the data point *k* as the exemplar for the data point *i*, based on the choice of other data points. At each iteration, these messages are updated as follows:

1 $$\;r(i,k)\leftarrow s(i,k)-\underset{k^{\prime }\;\;\text{s.t.}\;\;k^{\prime }\neq k}{\max}\left\{a(i,k^{\prime })+s(i,k^{\prime })\right\}$$

2 $$\;a(i,k)\leftarrow \min\left\{0,r(k,k)+\underset{i^{\prime }\;\;\text{s.t.}\;\;i^{\prime }\notin \{i,k\}}{\sum }\max\left\{0,r(i^{\prime },k)\right\}\right\},$$

where *s*(*i*,*k*) is the similarity between the data points *i* and *k*, used as the input of the clustering algorithm. This similarity *s*(*i*,*k*) can be asymmetric. The self-availability is updated in a slightly different way, as shown below:

3 $$a(k,k)\leftarrow \underset{i^{\prime }\;\;\text{s.t.}\;\;i^{\prime }\neq k}{\sum }\max\left\{0,r(i^{\prime },k)\right\}.$$

The data point *k* that maximizes the sum *a*(*i*,*k*)+*r*(*i*,*k*) is chosen as the exemplar for the data point *i*, and the algorithm converges if the set of exemplars does not change further.

So far, affinity propagation has been applied to various applications – such as predicting genes from microarray data and clustering facial images – and it has been shown to effectively identify meaningful clusters of data points at a much lower computational cost than traditional clustering methods [15]. One important advantage of affinity propagation is that the number of clusters need not be specified in advance. This is especially useful in our current application, since we neither know how many functional modules are embedded in the biological network at hand nor how many of them are relevant to cancer prognosis, which makes it practically difficult to determine how many subnetwork markers we should look for.

### 2.3 Computing the similarity between genes

In our proposed method, we use affinity propagation to identify clusters – or subnetworks – of discriminative and synergistic genes, whose protein products either interact with each other or are closely located in the PPI network. In order to use affinity propagation to identify the gene clusters, we first have to define the similarity *s*(*i*,*k*) between genes *g*_*i*_ and *g*_*k*_ for all gene pairs. The characteristics of the final clusters – especially, their usefulness as potential subnetwork markers – will critically depend on how we define this similarity. For this reason, we take the following points into consideration when defining *s*(*i*,*k*):

1. The proteins corresponding to the genes in the same cluster should have direct interaction or should be closely located in the PPI network.
2. Every gene in a potential subnetwork marker should have sufficient discriminative power to distinguish between the two class labels (metastatic vs. non-metastatic).
3. The discriminative power to distinguish between the two class labels should be increased by combining genes within the same cluster.

Based on these considerations, we define the similarity *s*(*i*,*k*) as follows:

4 $$s(i,k)=t_{k}+\min\left\{t_{\text{ik}}-t_{i},t_{\text{ik}}-t_{k}\right\}-\alpha \left|t_{i}-t_{k}\right|$$

if the shortest distance *d*(*i*,*k*) between the protein products of the genes *g*_*i*_ and *g*_*k*_ in the PPI network satisfies *d*(*i*,*k*)≤2. Otherwise, we set the similarity to *s*(*i*,*k*)=−*∞*. The discriminative power of a given gene is measured in terms of the *t*-test statistics score of the log-likelihood ratio (LLR) between the two class labels, and *t*_*i*_ and *t*_*k*_ are the *t*-test scores of *g*_*i*_ and *g*_*k*_, respectively. Similarly, *t*_*ik*_ is the *t*-test score of the combined LLRs of *g*_*i*_ and *g*_*k*_ which is computed by summing up the LLRs of the two genes. This term, *t*_*ik*_, reflects the discriminative power of the gene pair (*g*_*i*_,*g*_*k*_) after combining them. The self-similarity was set to *s*(*k*,*k*)=*c* for all *k*, where the constant *c* was chosen such that *s*(*i*,*k*)≥*c* for only 1% of all gene pairs (*g*_*i*_,*g*_*k*_). Uniform initialization of the self-similarity *s*(*k*,*k*)=*c* guarantees that every gene in the dataset gets equal chance to be an exemplar at the beginning of the message-passing process.

As shown in (4), the similarity *s*(*i*,*k*) between *g*_*i*_ and *g*_*k*_ is defined in an asymmetric way, where the first term corresponds to the discriminative power of the gene *g*_*k*_, the second term measures the improvement in discriminative power after combining the two genes *g*_*i*_ and *g*_*k*_, and the last term corresponds to a penalty term for the difference between *t*_*k*_ and *t*_*i*_. The parameter *α*∈ [ 0,1] is used to control the penalty term. According to the above definition, gene *g*_*i*_ regards gene *g*_*k*_ as being ‘similar’ to itself:

1. if *g*_*k*_ has high discriminative power (first term);
2. if combining the two genes increases the overall discriminative power;
3. if both genes have similar discriminative power.

The main reason underlying the asymmetric definition of the similarity *s*(*i*,*k*) is to indicate the direction of similarity. Based on our asymmetric definition, the exemplars of the identified clusters tend to have higher discriminative power compared to other non-exemplars. Intuitively, the gene similarity defined in (4) will make the affinity propagation algorithm identify gene clusters that consist of highly discriminative genes that are synergistic to each other and whose protein products are closely located in the PPI network.

### 2.4 Post-processing the identified gene subnetworks

Although the affinity propagation algorithm can effectively identify subnetworks that consist of discriminative and synergistic genes, the clustering process does not completely rule genes with relatively lower discriminative power out of those subnetworks. As a result, the initial subnetworks that are predicted by affinity propagation may still contain genes with relatively lower discriminative power compared to other genes in the same subnetwork. In order to improve the overall discriminative power of the potential subnetwork markers, we post-processed the initial subnetworks as follows. First, we clustered the genes in a given subnetwork into *k* groups based on their *t*-test statistics scores using the *k*-means clustering algorithm, where *k* was chosen to be *k*=⌊log(*#* of gene in considered subnetwork)+1⌋. After clustering, the genes in the group with the lowest average *t*-test score were removed from the subnetwork.

### 2.5 Probabilistic inference of subnetwork activity

For estimating the activity level of a subnetwork based on the gene expression profile of a patient, we adopted the probabilistic pathway activity inference method introduced in [4]. Given a subnetwork (or a pathway) with *n* member genes $G=\left\{g_{1},g_{2},\ldots ,g_{n}\right\}$ and the gene expression profile ***x***={*x*^1^,*x*^2^,…,*x*^*n*^} of a patient, where *x*^*i*^ is the expression level of the gene *g*_*i*_, the activity level of the subnetwork is computed by:

5 $$A(x)=\overset{n}{\underset{i=1}{\sum }}\lambda_{i}\left(x^{i}\right),$$

where *λ*_*i*_(*x*^*i*^) is the log-likelihood ratio between the two class labels (in this work, metastatic vs. non-metastatic). This is given by

6 $$\lambda_{i}(x^{i})=\log\left[\;\overset{1}{\underset{i}{f}}(x^{i})/f_{i}^{2}(x^{i})\right],$$

where $f_{i}^{j}(x^{i})$ is the conditional probability density function (PDF) of *x*^*i*^ under phenotype *j*. We assume that the gene expression level of *g*_*i*_ under phenotype *j* follows a Gaussian distribution.

## 3 Results

### 3.1 Statistics of the identified subnetwork markers

For each of the four datasets, we identified potential subnetwork markers using the proposed method and selected the top 50 markers based on their discriminative power, measured in terms of the *t*-test statistics score of the subnetwork activity. Three different values of *α* (=0.2,0.5,0.8) were used in our experiments to investigate the effect of the penalty term in (4) on the subnetwork marker identification result. Table 1 shows the average size of the top 50 subnetwork markers for each dataset and *α*. The last two columns in the table show the average size of the subnetwork markers identified using the method proposed by Chuang et al. [6], which we refer to as the ‘greedy’ method, for simplicity. Two different values of *r* were used for this greedy method. This parameter *r* specifies the minimum improvement rate of the discriminative power of a subnetwork marker. The greedy method stops when extending the subnetwork marker by adding a neighboring gene that does not improve the marker’s discriminative power by at least the specified rate *r*. We tested the greedy method with *r*=0.05 (or 5% minimum required improvement) which is the same as in [6]. We also tested the method with a lower rate *r*=0.001 (or 0.1% minimum required improvement) in order to allow the greedy search to continue even if the improvement is not very significant and find out how a lower rate affects the subnetwork size and its discriminative power. As we can see from Table 1, the size of the network decreases as *α* gets larger. In fact, a large *α* tends to cluster only genes with similar discriminative power (i.e., genes with similar *t*-test scores), thereby yielding smaller subnetworks with fewer genes. Similar trends can be also observed in Table 2, which shows the total number of unique genes in the top 50 subnetwork markers. As see can see in this table, a larger *α* results in a smaller number of unique genes in the top subnetwork markers, as each marker tends to get smaller.

**Table 1:** **Average size of the identified subnetwork markers**

Dataset	Proposed method			Greedy	
	***α***=0***.***2	***α***=0***.***5	***α***=0***.***8	***r***=0***.***05	***r***=0***.***001
USA	52.58	35.58	16.96	3.94	5.22
Netherlands	52.62	31.2	15.9	5.18	7.20
Belgium	37.64	20.2	12.3	4.12	5.48
Sweden	33.18	21.38	14.16	3.66	4.82

**Table 2:** **Total number of unique genes in the identified subnetwork markers**

Dataset	Proposed method			Greedy	
	***α***=0***.***2	***α***=0***.***5	***α***=0***.***8	***r***=0***.***05	***r***=0***.***001
USA	2,629	1,779	848	169	217
Netherlands	2,631	1,560	795	158	222
Belgium	1,916	1,010	615	113	149
Sweden	1,695	1,069	708	123	166

Table 3 shows the total number of the common genes between the identified subnetworks using different *α*. We can see that around 77% of genes included in the identified subnetworks using smaller *α* are also found in the subnetworks identified with larger *α*. We examined the overlap between the subnetworks identified on different datasets, which is defined as the number of genes in the intersection divided by the number of genes in the union. As shown in Table 4, we can see that the average overlap is typically close to (or above) 20%, which is larger than the greedy method as well as the overlap reported in [6] (12.7%).

**Table 3:** **Total number of common genes between the top subnetwork markers identified using different** ***α***

Dataset	***α***=0***.***2 ∩	***α***=0***.***2 ∩	***α***=0***.***5 ∩
	***α***=0***.***5	***α***=0***.***8	***α***=0***.***8
USA	1,612	660	561
Netherlands	1,382	646	488
Belgium	767	454	372
Sweden	802	466	387

**Table 4:** **Overlap between the top subnetwork markers identified on different datasets**

Dataset	Proposed method	Greedy	
	(***α***=0***.***5)	***r***=0***.***05	***r***=0***.***001
USA - Netherlands	25.10%	8.28%	7.60%
USA - Belgium	19.04%	5.22%	6.09%
USA - Sweden	19.71%	5.32%	5.51%
Netherlands - Belgium	18.11%	8.84%	10.09%
Netherlands - Sweden	18.85%	7.92%	7.78%
Belgium - Sweden	17.13%	11.57%	11.31%

### 3.2 Computational cost for subnetwork marker identification

**Figure 1:**
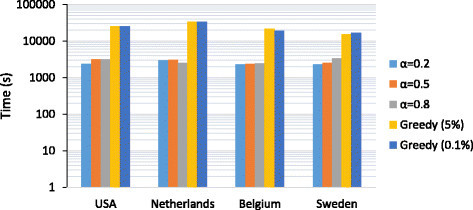
**Total CPU Time for identifying the top 50 subnetwork markers.** We evaluated the computational complexity of the proposed method by estimating the total CPU time needed for identifying the top 50 subnetwork markers in a given dataset. We compared our method with the previously proposed greedy method for a number of different parameters.

In order to evaluate the computational complexity of the proposed method, we computed the total CPU time that is needed for identifying the top 50 subnetwork markers on each dataset. We considered three different values of *α* (=0.2,0.5,0.8) that were used in our simulations. For comparison, we also estimated the total CPU time for the greedy method that was previously proposed. It should be noted that the two methods take completely different approaches for identifying multiple markers. In our proposed method, all potential subnetwork markers (whose total number exceeds 50) are *simultaneously* identified; hence, we need to rank the potential markers to select the top 50 markers with the highest discriminative power. As a result, for our proposed method, the total CPU time includes the time for calculating the similarity between genes, potential subnetwork marker identification through affinity propagation, and post-processing and ranking the subnetwork markers. On the other hand, for the greedy method, we measured the CPU time for calculating the discriminative power of the genes and iteratively searching for the top 50 markers. Since the greedy method finds one marker at a time, the search process needs to be repeated to find multiple markers. Figure 1 shows the total CPU time of the two methods for different parameters. All experiments were performed on a desktop computer with a 3.06 GHz Intel Core i3 CPU and 4GB 1333 MHz DDR3 memory. The results show that the proposed method is computationally more efficient for the given task as it can simultaneously identify all potential markers without repeating the search process multiple times. Unless one is interested in predicting only a few top markers, the proposed method provides a clear advantage over the previous greedy method. Figure 1 also shows that using different parameters does not affect the overall CPU time significantly.

### 3.3 Discriminative power of the subnetwork markers

**Figure 2:**
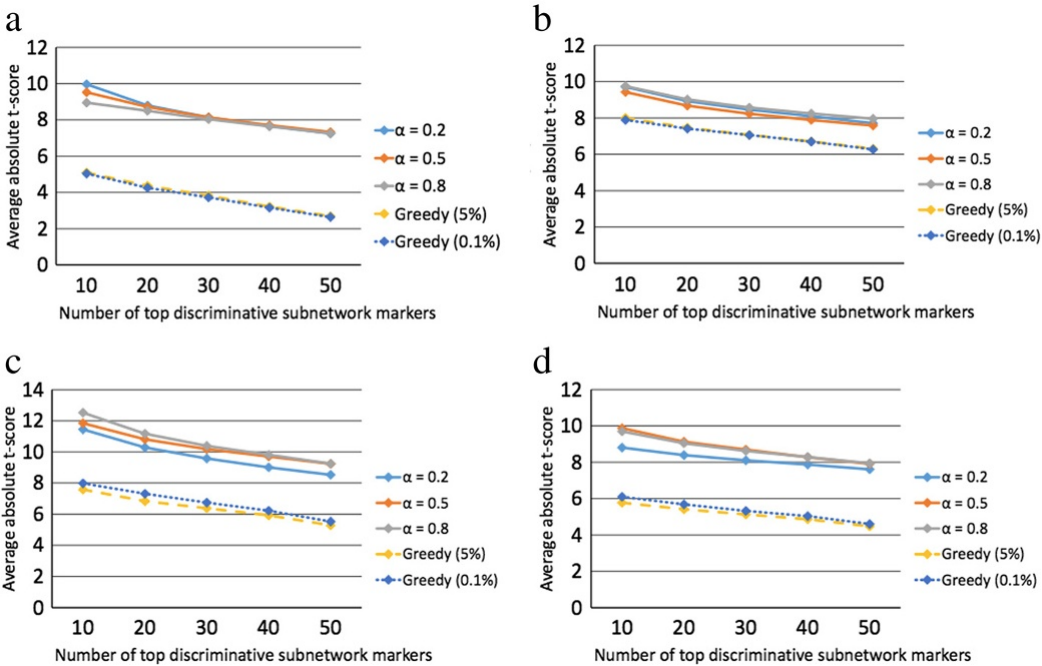
**Discriminative power of the identified subnetwork markers.** We computed the mean absolute *t*-test statistics score of the top *K* = 10, 20, 30, 40, and 50 subnetwork markers identified by different methods for the following datasets: **(a)** USA, **(b)** Netherlands, **(c)** Belgium, and **(d)** Sweden.

We evaluated the discriminative power of the predicted subnetwork markers by following a similar procedure as in previous studies [3],[4]. For each subnetwork marker identified using the proposed method, we first inferred its activity level for the gene expression profile of each patient and then computed the *t*-test score of the the inferred subnetwork activity level. Next, we sorted the subnetwork markers according to their absolute *t*-test score in a descending order. We then computed the average absolute *t*-test score of the top *K*=10,20,30,40,50 subnetwork markers, as shown in Figure 2.

The horizontal axis in Figure 2 corresponds to *K*, and the vertical axis corresponds to the mean absolute *t*-test score of the top *K* subnetwork markers. We compared the discriminative power of the subnetwork markers predicted by the proposed method with the discriminative power of the subnetworks predicted by the greedy method proposed in [6]. The activity level of these subnetworks (identified by the greedy method) was inferred based on the same scheme that was originally used in [6]. As we can see from Figure 2, the proposed method typically finds subnetwork markers with comparable or slightly higher discriminative power compared to the previous greedy method, although both methods work very well. In this experiment, the parameter *α* did not significantly affect the average discriminative power of the subnetwork markers identified by the proposed method.

We also investigated the impact of the post-processing step by comparing the discriminative power of the subnetwork markers before and after post-processing. Additional file 1: Figure S1 shows the results obtained using *α*=0.5. We can see that the discriminative power of the top 50 subnetwork markers improves as a result of the post-processing step, during which we remove the genes that have relatively lower discriminative power.

**Figure 3:**
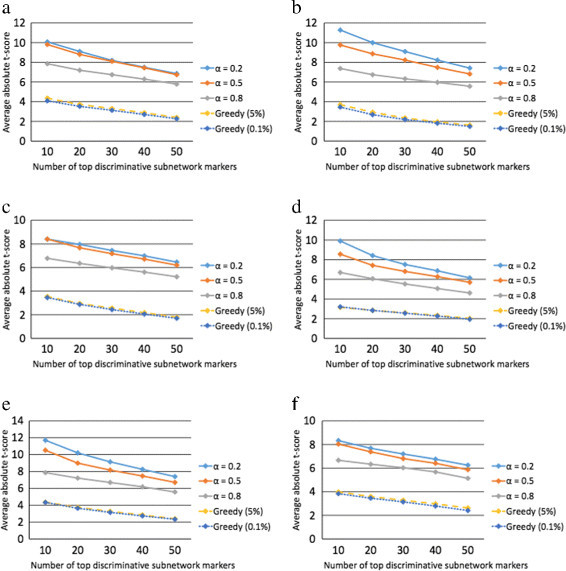
**Discriminative power of the identified subnetwork markers.** We computed the mean absolute *t*-score of the top *K* = 10, 20, 30, 40, and 50 markers for all datasets. The markers were identified using the first dataset and their discriminative power was evaluated on the second dataset. The experiments were performed for the following dataset pairs: **(a)** USA-Netherlands, **(b)** USA-Belgium, **(c)** USA-Sweden, **(d)** Netherlands-USA, **(e)** Netherlands-Belgium, and **(f)** Netherlands-Sweden.

Next, to test the reproducibility of the subnetwork markers identified by the proposed method, we performed cross-dataset experiments as follows. First, we identified subnetwork markers using the proposed method on one of the datasets and ranked the markers based on their absolute *t*-test statistics score. After ranking the subnetwork markers, we re-evaluated the discriminative power of the top 50 markers on a different dataset. This experiment allows us to find out how much discriminative power is retained by the top predicted markers in a different, and independent, dataset. The cross-dataset experiments are shown in Figure 3 and Additional file 1: Figure S2, where we can see that the markers identified by the proposed method remain highly discriminative across datasets. This is in clear contrast to the subnetwork markers identified by the greedy method [6], for which we can typically observe a sharp decrease in discriminative power when applied to an independent dataset that was not used for predicting the markers. Interestingly, we can also see that the proposed method finds effective markers that retain high discriminative power even on an independent gene expression dataset profiled on a different microarray platform. For example, in Figure 3a, the subnetwork markers were first identified using the USA dataset profiled on an Affymetrix chip and then evaluated on the Netherlands dataset profiled on a custom Agilent chip. Figure 3a shows that the markers predicted by the proposed method using the first dataset can also effectively discriminate between the two class labels based on the gene expression profiles in the second dataset. Similar trends can also be observed in Figure 3d,e,f and Additional file 1: Figures S2b,e.

One interesting observation we can make from these figures is that a smaller *α* tends to yield subnetwork markers that retain their discriminative power relatively better across independent datasets. This observation makes an intuitive sense, since a larger *α* tends to penalize genes with different discriminative power thereby giving rise to relatively smaller subnetwork markers that mostly consist of a few highly discriminative genes that may not be necessarily synergistic. This increases the risk of overfitting the data, thereby degrading the effectiveness of the predicted markers on other independent datasets.

### 3.4 Evaluating the reproducibility of the predicted subnetwork markers

In order to evaluate the efficacy of the predicted subnetwork markers in cancer prognosis, we performed five-fold cross-validation experiments based on a similar set-up that has been commonly used in previous studies [3]-[7].

Considering that our ultimate goal is to identify effective subnetwork markers that can be used for building robust classifiers that can accurately predict breast cancer prognosis, it is important to verify whether the predicted markers can actually lead to better classifiers whose performance can be reproduced on independent datasets. For this purpose, we performed the following cross-dataset experiments.

First of all, we selected one of the four breast cancer datasets just for identifying the potential subnetwork markers and selecting the optimal feature set (i.e., the set of markers to be used for building the classifier). To select the optimal set of features, we randomly divided the chosen dataset into three folds, where two folds (marker-evaluation set) were used for evaluating the discriminative power of the subnetwork markers and the remaining one fold (feature-selection set) was used for selecting the features to be used in the classifier. We used the entire set for estimating the class conditional probability density functions that are needed for the pathway activity inference [4].

We evaluated the discriminative power of all potential subnetwork markers based on the marker-evaluation set, selected the top 50 markers, and sorted them according to their absolute *t*-test score in a descending order. Initially, we built a classifier based on linear discriminant analysis (LDA), where only the top subnetwork marker was included in the feature set. The classifier was trained on the marker-evaluation set, and its classification performance was assessed by measuring the area under ROC curve (AUC) on the feature-selection set. Subsequently, we added the next best subnetwork marker to the feature set, re-trained and re-evaluated the classifier, and kept the subnetwork marker only if the AUC increased. We repeated this process for the top 50 subnetwork markers.

**Figure 4:**
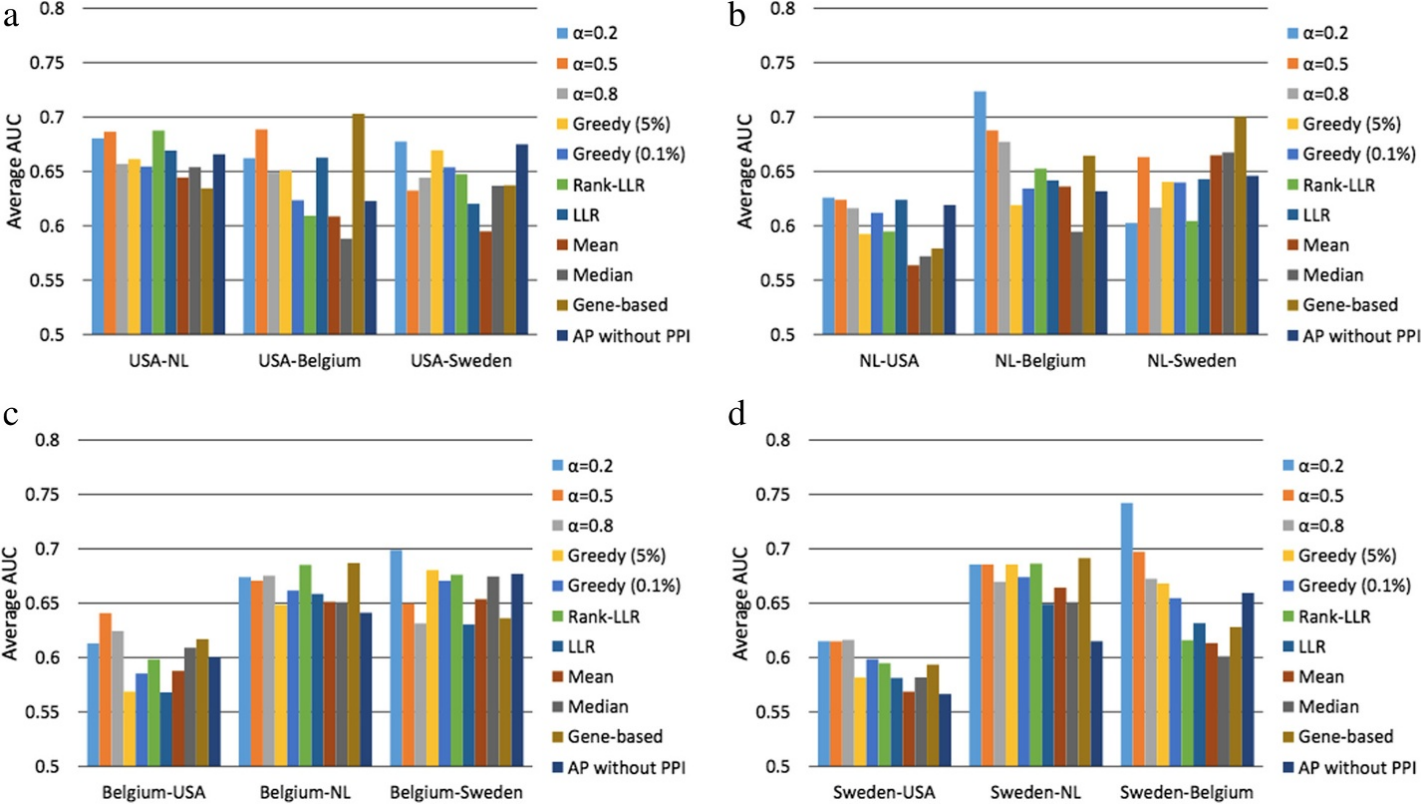
**Reproducibility of various subnetwork and pathway markers.** In order to evaluate the reproducibility of various modular markers, we used the first dataset to identify potential markers and select the optimal set of features and the second dataset to train the classifier (using the selected features) and evaluate its performance. Average classification performance is shown when the markers were selected based on **(a)** the USA dataset, **(b)** the Netherlands dataset, **(c)** the Belgium dataset, and **(d)** the Sweden dataset.

Next, we chose a different dataset to train an LDA classifier (using the markers selected from the first dataset) and evaluate its performance. For this, the second dataset was randomly divided into five folds, where four folds were used for training (without reselecting the features) and the rest was used for computing the AUC. The entire process was repeated for 100 random partitions, and we report the average AUC as the performance measure. Similar experiments have been performed to evaluate the classification performance of previous methods, including the greedy subnetwork marker identification method [6] as well as a number of pathway-based classification methods: Rank-LLR [5], LLR [4], Mean, and Median [2]. Each method uses a different way to infer the pathway activity level based on the expression levels of its gene members. For example, Mean (or Median) method uses the mean (or median) expression value of the member genes that belong to the same pathway. LLR and Rank-LLR both utilize the log-likelihood ratio between different phenotypes based on the expression level of each member gene. For pathway markers, we selected the top 50 pathways among the 880 pathways in the C2 curated gene sets in Molecular Signatures Database (MsigDB) [16]. Figure 4 summarizes the classification performance of different methods, where we can clearly see that the proposed method leads to more reliable classifiers with a much more consistent performance across different breast cancer datasets.

Finally, we also performed within-dataset experiments to investigate the performance of the proposed method and compare it with previous subnetwork and pathway-based methods. In these experiments, the classifiers were trained and evaluated on different folds of the same dataset, where a similar five-fold cross-validation set-up was used as before. We first selected a dataset and then randomly divided it into five folds. Four out of the five folds were used as a training set for building the classifier. The remaining one fold was used as a test set for evaluating the classification performance. The subnetwork markers were identified using the entire dataset, and not just the four fold training set, due to the high computational burden for re-identifying the subnetwork markers every time for a large number of random partitions. The results are depicted in Additional file 1: Figure S3. We can see that classifiers based on subnetwork markers performed significantly better compared to those based on pathway markers. The main reason for this significant performance improvement is the substantially increased coverage of genes, which was the main motivation for identifying subnetwork markers and using them for cancer classification. The proposed subnetwork marker identification method and the greedy method performed both well in the within-dataset experiments, although our proposed method outperformed the greedy method in terms of robustness and reproducibility across different datasets as we have shown before.

## 4 Conclusions

In this paper, we proposed a novel method for identifying robust and synergistic subnetwork markers that can be used to accurately predict breast cancer prognosis. Our proposed method utilizes an efficient message-passing algorithm called affinity propagation [15] to identify gene subnetworks that consist of discriminative and synergistic genes whose protein products are known to interact with each other or to be closely located in the protein-protein interaction network. The proposed method allows us to simultaneously identify multiple mutually exclusive subnetwork markers that have the potential to synergistically improve the prediction of breast cancer prognosis. Extensive evaluation based on four large-scale breast cancer datasets demonstrates that the proposed method can predict effective subnetwork markers with high discriminative power and reproducible performance across independent datasets. Furthermore, the predicted markers can be used to construct robust cancer classifiers that can yield more consistent classification performance across datasets compared to other existing methods.

## Additional file

## Electronic supplementary material

Additional file 1:**Supplementary material.** This supplement contains figures for additional experimental results. **Figure S1.** shows the discriminative power of the subnetwork markers identified by the proposed method with and without the post-processing step. **Figure S2.** contains charts showing the discriminative power of the subnetwork and pathway markers across different datasets. **Figure S3.** shows the classification results of the within-dataset experiments. (PDF 999 KB)

Below are the links to the authors’ original submitted files for images.

Authors’ original file for figure 1

Authors’ original file for figure 2

Authors’ original file for figure 3

Authors’ original file for figure 4

Authors’ original file for figure 5

Authors’ original file for figure 6

Authors’ original file for figure 7
