# Supplementary material for: Simultaneous identification of robust synergistic subnetwork markers for effective cancer prognosis
Source: EURASIP J Bioinform Syst Biol. 2014 Nov 6;2014:19. doi: 10.1186/s13637-014-0019-9 (PMC5270447; doi:10.1186/s13637-014-0019-9)
Supplement: Supplementary file 1 — Additional file 1:Supplementary material. This supplement contains figures for additional experimental results. Figure S1. shows the discriminative power of the subnetwork markers identified by the proposed method with and without the post-processing step. Figure S2. contains charts showing the discriminative power of the subnetwork and pathway markers across different datasets. Figure S3. shows the classification results of the within-dataset experiments. (PDF 999 KB) [file 13637_2014_19_MOESM1_ESM.pdf]

Additional File 1: Supplementary Material for "Simultaneous Identification of Robust Synergistic Subnetwork Markers for Effective Cancer Prognosis"

Navadon Khunlertgit[1] and Byung-Jun Yoon[1,2,*]

[1] Department of Electrical and Computer Engineering, Texas A&M University, College Station, TX 77843-3128, USA

[2] College of Science, Engineering, and Technology, Hamad Bin Khalifa University (HBKU), P.O. Box 5825, Doha, Qatar

[*] Correspondence: Byung-Jun Yoon (bjyoon@ece.tamu.edu)

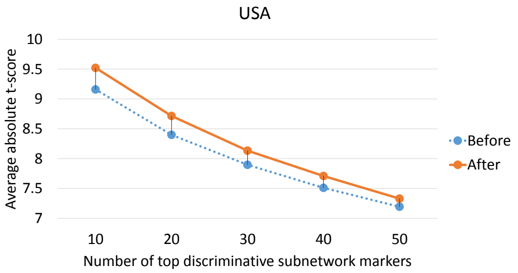

(a)

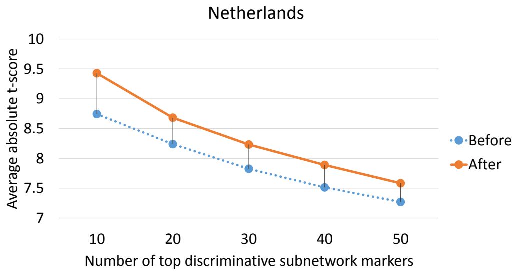

(b)

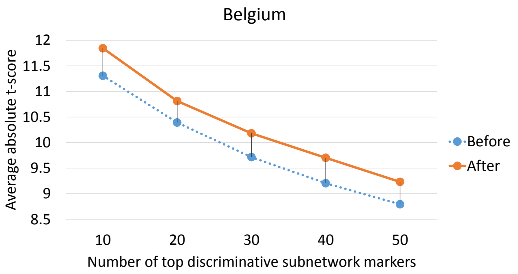

(c)

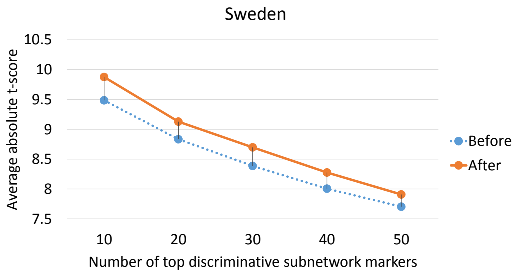

(d)

Figure S1: **Discriminative power of the identified subnetwork markers.** We computed the mean absolute t-score of the top K=10, 20, 30, 40, and 50 markers identified by the proposed method with and without post processing step for the following datasets: (a) USA, (b) Netherlands, (c) Belgium, and (d) Sweden.

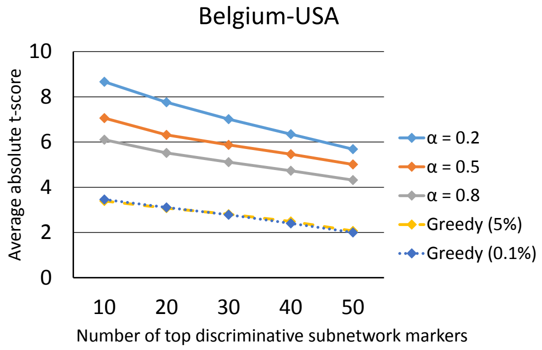

(a)

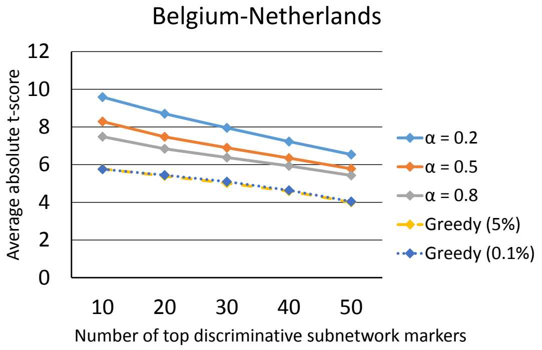

(b)

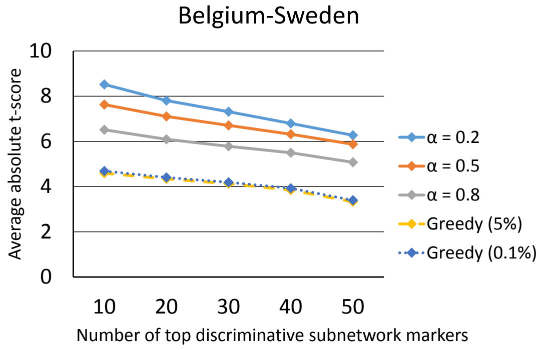

(c)

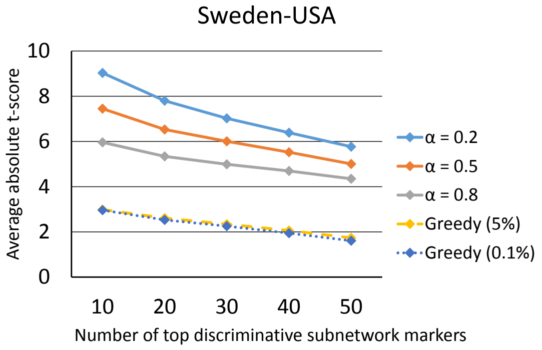

(d)

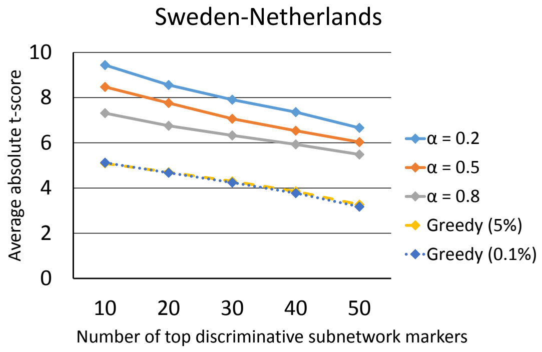

(e)

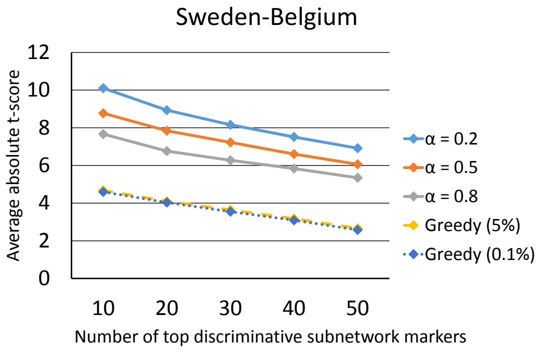

(f)

Figure S2: **Discriminative power of the identified subnetwork markers.** We computed the mean absolute t-score of the top K=10, 20, 30, 40, and 50 markers for all datasets. The markers were identified using the first dataset and their discriminative power was evaluated on the second dataset. The experiments were performed for the following dataset pairs: (a) Belgium-USA, (b) Belgium-Netherlands, (c) Belgium-Sweden, (d) Sweden-USA, (e) Sweden-Netherlands, and (f) Sweden-Belgium.

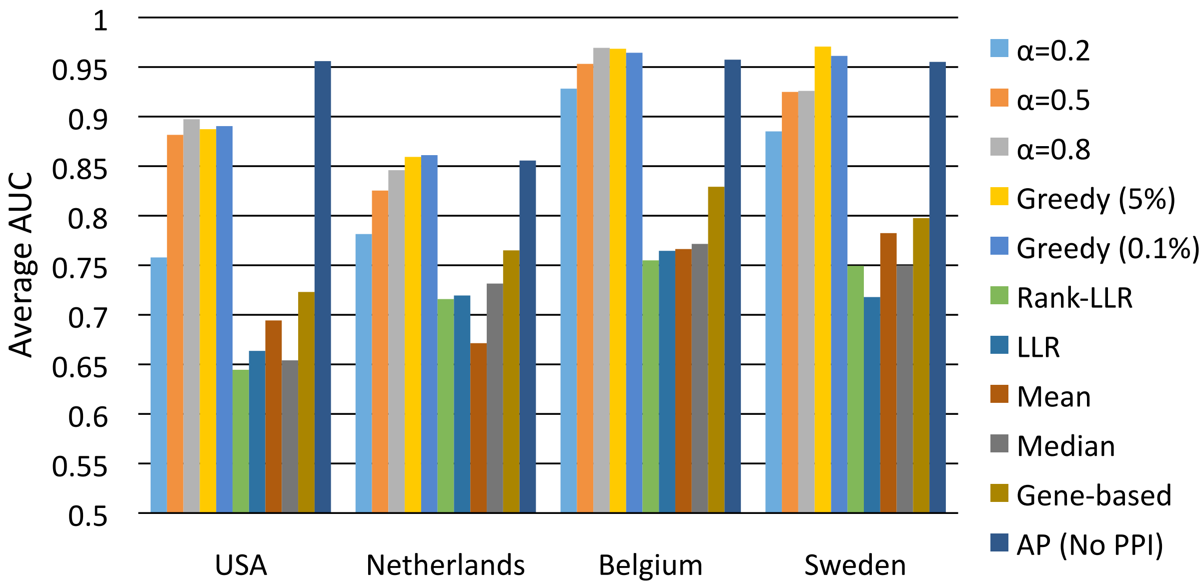

Figure S3: **Classification performance.** We performed cross-validation experiments to evaluate the classification performance of several subnetwork and pathway marker identification methods. The marker were identified using the entire dataset. The classifiers were trained and evaluated on different folds of the same dataset.
