# Supplementary figures and images for: Simultaneous identification of robust synergistic subnetwork markers for effective cancer prognosis
Source: EURASIP J Bioinform Syst Biol. 2014 Nov 6;2014:19. doi: 10.1186/s13637-014-0019-9 (PMC5270447; doi:10.1186/s13637-014-0019-9)

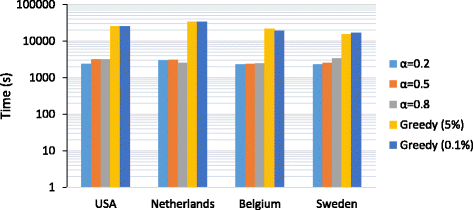

Supplement: Supplementary file 2 — Authors’ original file for figure 1 [file 13637_2014_19_MOESM2_ESM.gif]

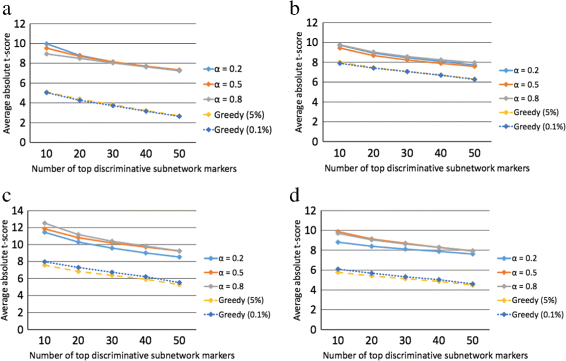

Supplement: Supplementary file 3 — Authors’ original file for figure 2 [file 13637_2014_19_MOESM3_ESM.gif]

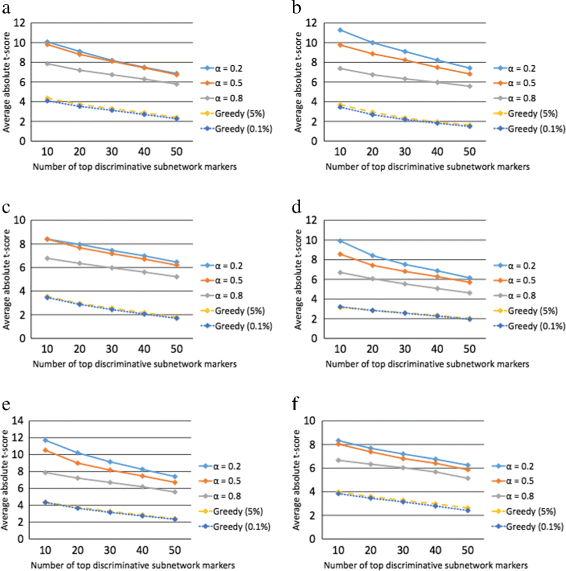

Supplement: Supplementary file 4 — Authors’ original file for figure 3 [file 13637_2014_19_MOESM4_ESM.gif]

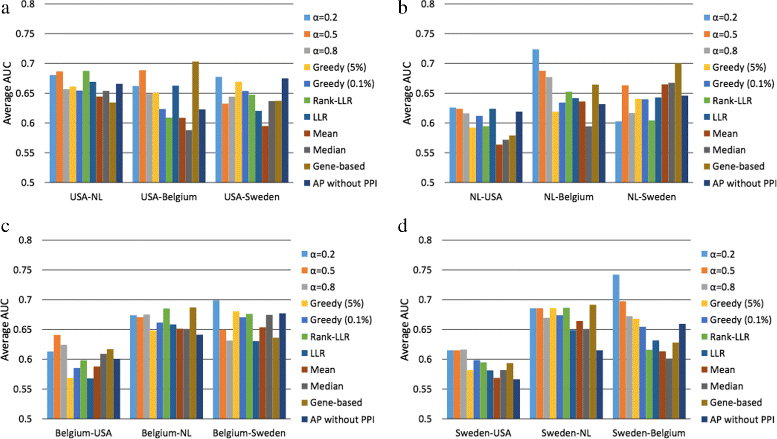

Supplement: Supplementary file 5 — Authors’ original file for figure 4 [file 13637_2014_19_MOESM5_ESM.gif]

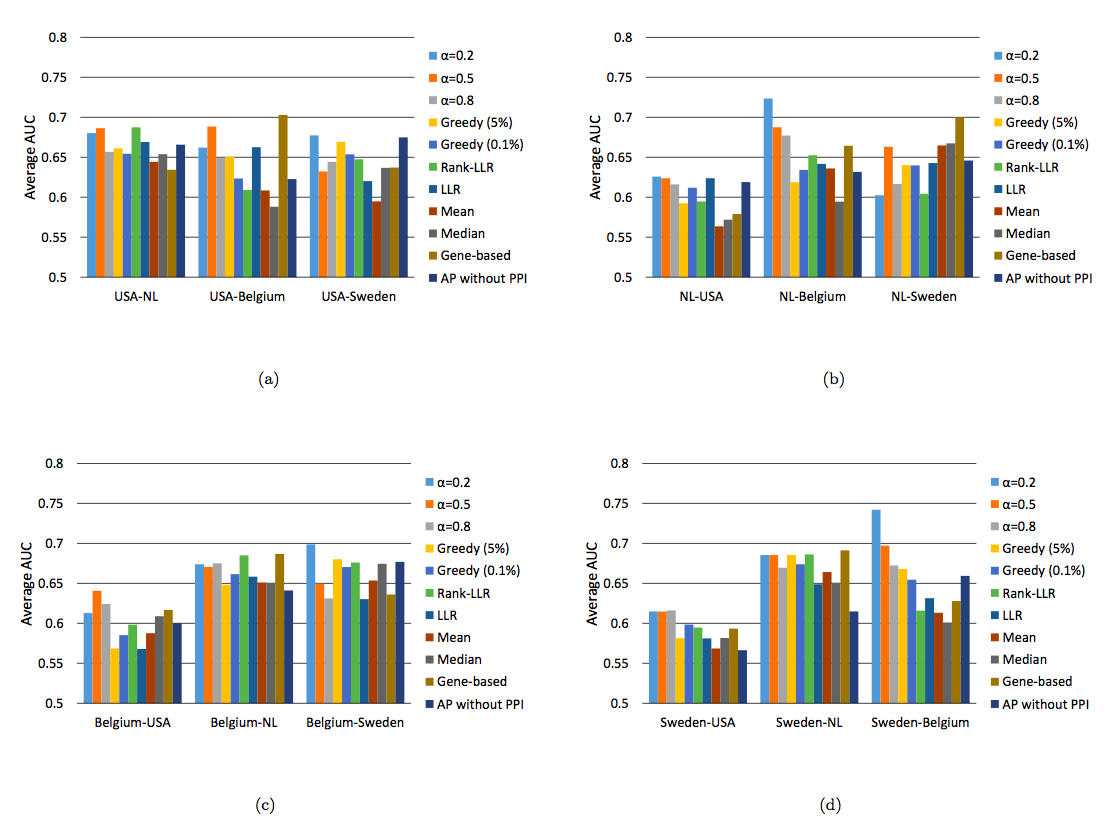

Supplement: Supplementary file 6 — Authors’ original file for figure 5 [file 13637_2014_19_MOESM6_ESM.png]

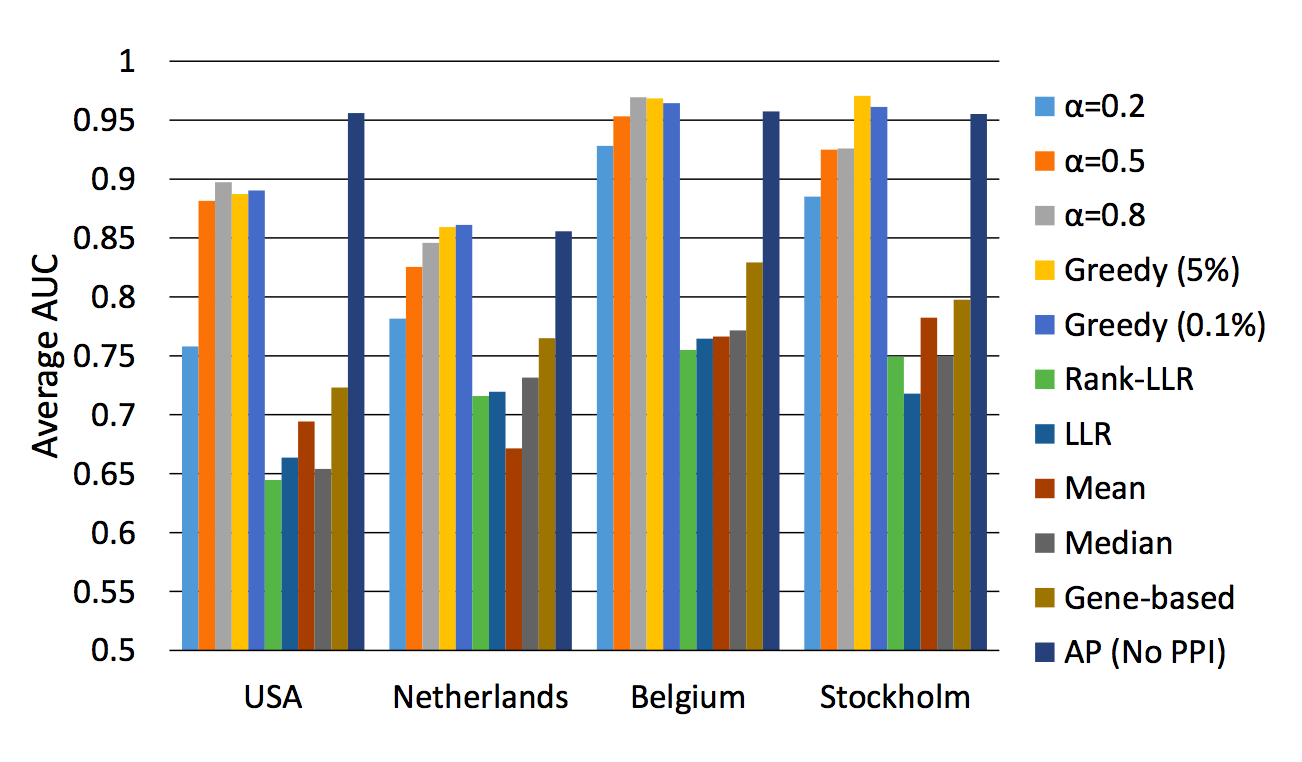

Supplement: Supplementary file 7 — Authors’ original file for figure 6 [file 13637_2014_19_MOESM7_ESM.png]

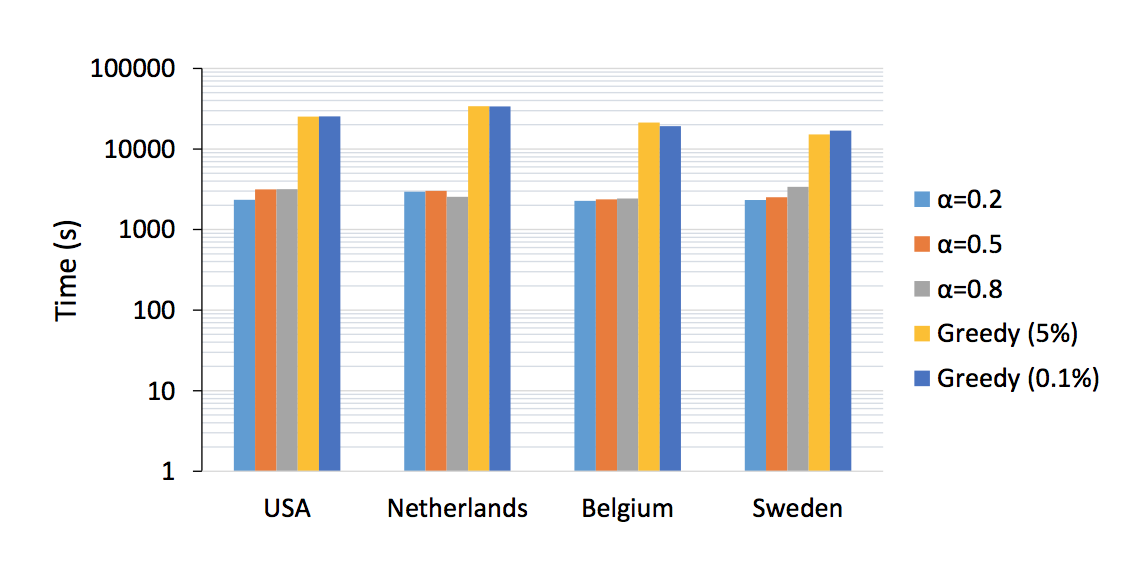

Supplement: Supplementary file 8 — Authors’ original file for figure 7 [file 13637_2014_19_MOESM8_ESM.png]
